# The Moral Psychological Justification of Anger: An Exploration of Self-Respect and Recognition

**DOI:** 10.3390/bs15010003

**Published:** 2024-12-24

**Authors:** Jinjin Zhang, Zhiheng Xiong, Hao Zheng, Xiangzhen Ma

**Affiliations:** School of Humanities, Southeast University, Nanjing 211189, China; 230189865@seu.edu.cn (J.Z.); xzh_psy@seu.edu.cn (Z.X.); 230219059@sed.edu.cn (H.Z.)

**Keywords:** anger, self-respect, recognition, moral value

## Abstract

In the field of moral psychology, traditional perspectives often evaluate anger based on its consequences, either validating or condemning it for its perceived benefits or harms. This paper argues for a shift in focus from the outcomes of anger to its moral and psychological foundations. By integrating insights from psychological research, this study posits that the fundamental nature of anger is intrinsically linked to the quest for recognition. Justified anger is defined as an emotional response to the unmet need for fair acknowledgment, while unjustified anger stems from feelings of superiority and the pursuit of higher status. This paper distinguishes between these two forms of anger, providing a more nuanced and intuitive understanding of the emotion. This interpretive framework not only aligns with our experiential understanding of anger but also offers a theoretical bridge to reconcile divergent philosophical and psychological perspectives. This study emphasizes the importance of addressing the underlying issues of recognition and self-esteem, suggesting that anger, when properly understood and managed, can serve as a constructive force for social justice and mutual respect.

## 1. Introduction

Typically, anger is regarded as an irrational and destructive emotion that should be eliminated. For instance, Seneca argues that anger is the most dreadful and absurd of all emotions, leading even the avenger to self-destruction (Kaster & Nussbaum, 2010). Nussbaum contends that anger is counterproductive as it is driven by a desire for “payback”, indicating distorted moral priorities and irrational beliefs (Nussbaum, 2016). She posits that such sentiments hinder genuine moral advancement in response to wrongdoing and harm. Similarly, Pettigrove emphasizes the importance of avoiding anger, even in cases of political injustice, as anger can potentially dismiss likely allies, escalate conflict, and hinder the pursuit of justice (Pettigrove, 2012).

By contrast, some philosophers advocate the moral value of anger, arguing that it can serve as a powerful motivator for individuals to address injustices and strive for social fairness. For instance, Tessman argues that although the anger of the oppressed cannot be considered “good” in the Aristotelian sense due to its lack of the moderation necessary for flourishing, it can still be viewed as “good” in terms of its long-term consequential significance—specifically in promoting the well-being of others (Tessman, 2005). McBride suggests that anger is a virtue when channeled effectively and can be constructive in addressing social injustices and empowering marginalized communities (McBride, 2018). Similarly, Cherry contends that anger is vital in combating racial injustice (Cherry, 2021). Wallaert explores the concept of feminist anger in the context of movements such as #MeToo and Women’s Marches, emphasizing paying attention to feminist anger, both within oneself and others, as crucial to achieving epistemic equality (Wallaert, 2023). Nonetheless, some philosophers argue that anger, even if counterproductive, can still be considered a fitting response to the state of the world (Bell, 2009; Callard, 2017; Srinivasan, 2018). The debate on the moral value of anger often focuses on its consequences. Despite the positive or negative outcomes of anger, simply contemplating the benefits of anger does not justify it and considering the drawbacks of anger does not eliminate the feelings.

In addition to these philosophical perspectives, psychological research has provided valuable insights into the nature and functions of anger. Lazarus and Oatley and Jenkins present anger as a basic emotion with important adaptive functions, such as motivating individuals to solve problems, overcome obstacles, or express disagreement with the breaking of rules, including moral ones (Lazarus, 1991; Oatley et al., 2006). Novaco and Spielberger et al. highlight the role of anger in emotional regulation and its potential to drive constructive action (Novaco, 2010; Spielberger, 1995). Berkowitz and Wranik and Scherer further elaborate on the cognitive and physiological aspects of anger, emphasizing its role in signaling and responding to perceived threats (Berkowitz, 2012; Wranik & Scherer, 2010). Lewis discusses the developmental and social dimensions of anger, underscoring its role in interpersonal relationships and social interactions (Lewis, 2010). While psychological research has made significant contributions to our understanding of anger, it often focuses on its adaptive functions, emotional regulation, cognitive and physiological aspects, and developmental and social dimensions. These perspectives, however, may not fully address the moral and psychological foundations of anger, the distinction between justified and unjustified anger, and the complex interplay between anger, self-esteem, and recognition. By integrating both philosophical and psychological perspectives, our study aims to provide a more nuanced and comprehensive understanding of anger, highlighting its potential as a morally and psychologically significant emotion.

This study aims to defend the justification of anger. Unlike the aforementioned defense, our focus is not on the positive social outcomes of anger but on its moral and psychological foundations. Building upon relevant psychological studies, we argue that the desire behind anger is for recognition, rather than revenge. Srinivasan mentions this perspective does not offer a thorough argument (Srinivasan, 2018). Similarly, Silva supports this view (Silva, 2021), refuting Nussbaum’s assertion that anger is “retributive” and “ineffective” (Nussbaum, 2016). Silva argues that the desire behind anger is for recognition, not for revenge, in defense of the moral value of anger. However, Silva does not explain why the desire for recognition justifies the moral value of anger. We complement Silva’s argument by contending that recognition justifies the desire behind anger by elucidating the relationship between anger, self-esteem, moral injury, and recognition. Additionally, we distinguish between justified and unjustified anger, explaining that justified anger is an emotional response based on an unfulfilled desire for equal recognition, whereas unjustified anger stems from an unmet desire for recognition of superiority, leading to feelings of arrogance. Finally, we argue that the central issue when considering anger is not its consequences, but rather the personal and social issues revealed through it. Therefore, we should focus on resolving conflicts and transforming anger into a constructive force that fosters mutual understanding, recognition, and respect among individuals and groups.

This study posits that anger can be justified based on the desire for recognition. Anger not only serves as a valid emotional response but also highlights instances where it is unwarranted. Specifically, justified anger arises from an unfulfilled desire for equal recognition, while unjustified anger stems from a quest for superiority and is often accompanied by arrogance. This perspective not only aligns more intuitively with our understanding of anger but also provides a framework to reconcile conflicting viewpoints. By distinguishing between these two forms of anger, this study offers a nuanced approach that emphasizes the importance of addressing the underlying issues of recognition and self-esteem, thereby fostering a more constructive and empathetic engagement with this complex emotion.

## 2. The Claim of “Retaliation” and “Inefficacy”

### 2.1. The Retaliatory Nature and Inefficacy of Anger

According to the Orthodox perspective, anger is generally discouraged due to its tendencies toward retaliation and the negative consequences associated with it. Nussbaum prominently represents this viewpoint (Nussbaum, 2016), arguing that anger is inevitably linked to retaliation: “anger involves, conceptually, a wish for things to go badly, somehow, for the offender, in a way that is envisaged, somehow, however vaguely, as a payback for the offense”. While Nussbaum acknowledges that anger may be triggered by unfair treatment, she maintains that anger is ineffective in the struggle for social justice, as it often comes with retaliation, which can lead to destructive outcomes.

According to Nussbaum (Nussbaum, 2016), the inefficacy of anger manifests in two aspects: from an individual perspective, anger not only fails to compensate for the specific losses of the victim but also cannot restore the dignity and worth of the victim; and anger cannot promote social fairness and justice in the society. In the case of the former aspect, regarding the view that anger rises in response to humiliation and degradation, Nussbaum considers this “road of status” to be a “narcissistic fallacy”, as “the tendency to see everything that happens as about oneself and one’s own rank seems very narcissistic and ill-suited to a society in which reciprocity and justice are important values. This view ignores the sense that actions have an intrinsic moral worth: that rape is bad because of the suffering it inflicts, and not because of the way it humiliates the friends of the victim”. In Nussbaum’s view, wrongful behavior harms specific elements such as health, safety, physical integrity, and finances. However, revenge does not compensate for these losses. Moreover, Nussbaum considers two other situations, namely, the harm caused by sexual assault and racial and gender discrimination. Regarding the former, she believes that sexual assault not only damages physical integrity but also dignity. Nussbaum states that even if the dignity of the offender is diminished, the victim’s respect cannot be elevated. For the latter, Nussbaum argues that the need “is equal respect for human dignity. What is wrong with discrimination is its denial of equality, as well as its many harms to well-being and opportunity. Reversing positions through down-ranking does not create equality. It just substitutes one inequality for another” (Nussbaum, 2016). In other words, irrespective of the extent to which a victim’s personal dignity has been diminished, anger cannot restore dignity or compensate for its loss.

The second aspect holds that anger cannot promote social fairness and justice in the society. Nussbaum asserts that anger is fundamentally aimed at retaliation rather than reconciliation, and that retaliatory actions pose the risk of being counterproductive (Nussbaum, 2016). She cites examples of successful social movement leaders, such as Martin Luther King and Mandela, who achieved victories through non-anger, non-violent approaches in their pursuit for a just society, emphasizing cooperation, generosity, and collaboration.

Pettigrove endorses Nussbaum’s stance and opines that anger is a defense response system based on the “smoke detector” principle (Pettigrove, 2012). Specifically, anger functions as a defense mechanism that triggers exaggerated responses to potential threats, leading individuals to focus on hostile stimuli rather than accurately assessing their surroundings. This perceptual distortion can result in individuals attributing greater blame to others for negative events, perpetuating a cycle of escalating anger and a heightened sense of responsibility for those events. Therefore, anger affects people’s risk assessment (Pettigrove, 2012).

In conclusion, the primary argument against anger posits that it inherently involves desires for retaliation and punishment. Consequently, anger is perceived as the inability to restore the dignity and worth of the victim on an individual level and the incapability to promote social fairness and justice on a societal level.

### 2.2. Rebuttal of the Claim of “Retaliation” and “Inefficacy”

Seneca and Nussbaum, along with other philosophers, deny the value of anger for a common reason: anger entails retaliation. Most scholars oppose Nussbaum’s claim that anger is ineffective, arguing that anger is effective in promoting social justice and fairness (Tessman, 2005; McBride, 2018; Cherry, 2021; Wallaert, 2023; Leboeuf, 2018). Some scholars suggest that anger is still a normal reaction to injustice even though it may be ineffective in promoting social justice (Bell, 2009; Callard, 2017; Srinivasan, 2018). Additionally, only a few scholars have refuted the retaliatory theory of anger (Silva, 2021). We primarily question the premise of the retaliatory theory and argue that the motivation behind anger is not revenge but rather recognition. This interpretation is appealing as it aligns with our intuition and reconciles opposing viewpoints.

McBride and Leboeuf defend the moral value of anger in their dialogues with Nussbaum (McBride, 2018; Leboeuf, 2018). Nussbaum advocates a transitional anger (Nussbaum, 2016); for instance, anger generated by non-white Americans toward racist policies and institutions should be “purified” to transform inner anger into love and generosity, striving to win the friendship and cooperation of the other party. In her view, wrongdoers should be spared from bowing and humiliation, as this will not correct any mistakes or bring us closer to justice. However, McBride criticizes Nussbaum’s concept of transitional anger as callous and insensitive to those who suffer unjust injury (McBride, 2018). Anger is a response and resistance to the perpetrator’s infringement, which helps alleviate the victim’s feelings of being harmed or, at least, changes the conditions that allow the impact of the harm. Anger helps maintain a sense of being mistreated, achieving a therapeutic release for the victim and enabling them to remain vigilant against danger. Therefore, anger is a necessary and appropriate response to unfair harm, as perceived by victims. This anger gives people the courage to confront and resist unjust harm, thus enabling improvements in unjust situations.

Similarly, Leboeuf challenges Nussbaum’s assertion regarding the inefficacy of anger, arguing that anger is crucial in combating social oppression, particularly racial discrimination (Leboeuf, 2018). Leboeuf illustrates a scenario in which black individuals are subjected to the scrutinizing gaze of white individuals, leading to feelings of disorientation and physical impairment, termed “bodily alienation”. In this context, anger functions as a shield for the marginalized group against the harm inflicted by the oppressor’s gaze. In situations of racial oppression, whereby the oppressed are dehumanized, anger serves as a tool to deepen their comprehension of the oppressors and prompts introspection within the oppressed community. Ultimately, anger emerges as a form of resistance against prejudice. When confronting scrutiny of those in power, anger can inspire the oppressed to confront the hostility stemming from discriminatory attitudes. However, defending anger solely from the standpoint of its outcomes can lead both sides of the divide into an endless empirical debate, as anger can have destructive consequences as well as positively promoting social fairness and justice.

Certainly, some scholars still justify anger despite its potential negative consequences, because anger is sometimes an appropriate response to an unjust world. For instance, Srinivasan suggests that “whether anger is an apt or fitting response to the world, does not turn on the consequences, good or bad, of that anger. Apt anger can be counterproductive, making the angry person worse off and exacerbating the situation at which the person is angry. It is plausible that this is especially true for victims of systematic injustice whose apt anger at their oppression may invite further violence and retrenchment” (Srinivasan, 2018). She distinguishes between intrinsic and instrumental reasons, suggesting that scholars who believe that anger has a counterproductive effect face a dilemma: it is challenging to explain why instrumental reasons should take precedence over intrinsic reasons when the two types of reasons come into conflict. Furthermore, Srinivasan believes that this conflict in which oppressed victims must choose between fitting anger and cautious action constitutes an unidentifiable form of injustice, known as emotional injustice. However, Srinivasan also experiences the dilemma of determining the standard by which to measure emotional justice, as it is evident that not all anger is justified.

Next, we focus on refuting the claim of “retaliation”, as Nussbaum opposes anger primarily on the grounds that it often leads to retaliation, which is harmful to both individuals and society. We posit that the desire for anger is not for revenge, but for recognition.

Some scholars argue that anger, a potent and readily triggered emotion, significantly contributes to violent and aggressive behaviors (Novaco, 2010). Although anger, hostility, and aggression share overlapping definitions, equating anger with retaliation oversimplifies their complex interrelations (Spielberger, 1995). It is also crucial to differentiate anger from hatred. While anger is a fleeting emotional response, hatred embodies a persistent, antisocial attitude marked by cognitive distortions and impaired emotional processing (Gawda, 2023). Anger protests perceived wrongs, while hatred seeks harm or destruction (Brudholm, 2010).

Research on psychology suggests no inherent link between anger and revenge. While many philosophers believe that angry people tend to attack, humiliate, or retaliate against those who behave immorally (Kaster & Nussbaum, 2010; Nussbaum, 2016; Cope & Sandys, 2010), psychologists such as Averill demonstrate that anger is an aggressive impulse but does not necessarily lead to aggressive behavior (Averill, 1983). Such impulse can be controlled, transformed, displaced, or sublimated. Similarly, Shoemaker suggests that although anger tends to be majorly retaliatory, its fundamental behavioral goal is to express anger, not just to retaliate against the offender (Shoemaker, 2018). Early psychological research also supports this conclusion. Some psychologists have conducted a thorough investigation of subjects’ various emotion-related action tendencies, actions, and goals, finding that two action tendencies were most closely related to anger: “wanting to hit someone” and “wanting to yell loudly”, and “saying some vulgar words” and “wanting to hurt someone” (Roseman et al., 1994). These two action tendencies share a common characteristic, namely, conveying anger to the offender. For avengers, the goal of conveying a message to the wrongdoer is more important than just seeing the wrongdoer suffer (Gollwitzer & Denzler, 2009; Gollwitzer et al., 2011). The fundamental purpose of anger is to communicate.

Some empirical studies in psychology suggest that anger correlates positively with “constructive behavior”. Psychologists such as Tausch et al. conducted a survey regarding the mandatory increase in tuition fees for German university education in real life (Tausch et al., 2011). Respondents were asked to indicate their likelihood of participating in 16 different types of anti-tuition fee actions in the future. These actions could be categorized into three types: “constructive actions”, including distributing flyers, signing petitions, and participating in demonstrations; “destructive actions”, such as setting fire to university buildings or private property; and “intermediate actions”, which involve disruptions such as blocking university buildings or public roads. Generally, individuals who oppose expressing anger surmise that anger will primarily lead to “destructive actions” or “intermediate actions”. However, contrary to common expectations, the research results indicate that anger is positively correlated with “constructive behavior” (Tausch et al., 2011). Therefore, anger is not always accompanied by destructive actions.

The desire behind anger primarily entails seeking recognition and respect rather than exacting revenge. While many believe that anger is caused by pain, in reality, pain is merely a physiological sensation. Anger is directed at others’ disrespect and belittling attitudes toward oneself. For instance, if someone steps on “my” foot intentionally, it may make “me” very angry, whereas if it was unintentional, the anger would be greatly reduced or even disappear, despite the pain being identical. Therefore, anger is not triggered by feelings of pain, but rather by others’ disrespect and belittlement toward oneself or loved ones, which can also be described as a feeling of being offended.

In behavioral sciences, feelings of offense are closely linked to a sense of humiliation and breaches of personal dignity (Tavuchis, 1993). Empirical research supports this view. Lemay, Overall, and Clark found that individuals feeling neglected or harmed often communicate their distress—such as through a cold shoulder or verbal complaints—before considering retaliation. This approach seeks acknowledgment and resolution (Lemay et al., 2012). Tangney, Stuewig, and Mashek similarly observed that angry individuals prefer to seek justice and respect over vengeance. Sincere apologies and efforts to resolve conflicts are more likely to reduce negative emotions and restore trust (Tangney et al., 2007). For example, a spouse’s prolonged neglect can provoke anger in their partner, perceived as an affront to their well-being and self-worth. However, initial responses rarely involve direct retaliation. Instead, the aggrieved partner may express distress through subtle or overt communication, such as withdrawal or verbal complaints (Lemay et al., 2012).

In conclusion, viewing anger as predominantly retaliatory and harmful is overly simplistic and overlooks its role in human interactions. Research indicates that while anger can drive aggressive impulses, it is far from being a direct path to destructive behavior. Anger often functions as a communicative tool, signaling the need for recognition and respect. Empirical evidence suggests that anger can foster constructive behaviors, such as addressing injustices and advocating change, rather than merely inciting retaliation. It primarily signals boundary violations and demands redress, catalyzing interpersonal and societal progress. Recognizing its protective and communicative aspects challenges the notion that anger should be suppressed entirely.

## 3. Anger, Self-Respect, Moral Injury, and Recognition

People feel angry primarily due to the threat to their self-respect, not just because their bodies or minds are threatened or harmed. If the threat or harm is caused by bad luck or natural disasters, we may feel sad, but not angry. Similar to anger, sadness also focuses on loss, which is painful. However, sadness centers on the event, such as death or disaster, which may be as a result of someone’s actions or an act of nature. As the focus remains on the loss caused by this event, even if an event is considered to be caused by someone, the person is not the target. Therefore, the concept of “wrongfulness” is irrelevant to sadness because loss is experienced, irrespective of whether or not it is due to a mistake (Nussbaum, 2016). People get angry because they feel morally harmed. Philosophers who deny the legitimacy of anger do not consider “humiliation” or “belittlement” as legitimate reasons for anger, but agree that anger is a response to moral harm.

When faced with natural harm, people may feel fear rather than anger. Both fear and anger stem from the threat posed to someone or something we value. However, compared to fear, anger does not merely concern the threat to what we value, but also a sense of malice. If a person lives isolated in a natural world that poses a threat to them, holds no superstitious or religious beliefs about how the world operates, and has no intentional human aggressors in this world, then they may experience fear without anger. As will be discussed, even the “original people” used self-defense weapons against wild animals, but lacked anger because they did not perceive the harm they suffered as intentional or as punishment. Therefore, intentional rather than natural harm causes people to feel anger. Let us compare two scenarios: a person approaches you and steps on your foot; and a person does not notice you next to them, accidentally steps on your foot, and then apologizes to you. Assuming that the pain caused in both situations is the same, which scenario would make you react with anger? Obviously, the first scenario because it reflects the indifference and malice of others toward us.

Intentional harm also conveys the idea that a person is not accorded due value or is unentitled to fair treatment; this devalues a person’s worth. Anger is not only an emotional protest against derogatory behavior, but also a defense of one’s own value and self-respect. Self-respect refers to seeing oneself as an equal individual; therefore, people with strong self-respect will neither be enslaved nor remain silent after being degraded. Allowing criminals to go unpunished equates to treating victims as less valuable. As Murphy suggests, the main value defended by righteous anger is self-respect, appropriate self-respect is fundamentally linked to anger, and a person’s dissatisfaction with the mental harm they have suffered stems from a concern for their own moral worth (Murphy, 1988). By contrast, if a person has low self-worth, they may not experience anger in response to abusive behavior. For instance, a female slave who endures mistreatment from her master without resistance may have internalized a diminished sense of self-worth. This low value in terms of her bodily integrity prevents her from feeling anger in response to the harm inflicted on her.

Some may argue that if a person confirms and acknowledges their own worth, they will be unafraid of being devalued because this does not necessarily mean that their true value will be lowered unless they lack confidence in their self-worth. This implies that people feel that their status and value have been diminished because they lack self-assurance. In other words, if a person is certain of their own worth, they will not feel hurt. On the one hand, no one can truly confirm their own value in the absence of others. Acknowledging oneself as second person requires confronting the perceptions of others. It is through others’ reactions to oneself that one can see oneself clearly. On the other hand, as Murphy states, “Some weaknesses and vulnerabilities in the area of self-esteem seem to me an ineliminable part of the human condition” (Murphy, 1988). It is impossible for a person to completely disregard the thoughts of others. While a person may be strong enough to resist attacks on their self-esteem, in reality, no one is powerful enough to be entirely indifferent to other people’s criticisms. As a relational being, to validate one’s values and dignity inherently necessitates recognition from others.

Furthermore, anger is closely related to moral harm, which primarily entails the lack of recognition and respect in “self-relation”; “self-relation refers to the consciousness or feeling that a person has about himself or herself with regard to the capabilities and rights they enjoy” (Honneth & Farrell, 1997). Honneth differentiates three levels of self-relationship, each corresponding to one of three types of moral injury. The first level of self-consciousness entails recognizing one’s body and desires as essential components of oneself. At this stage, moral harm manifests as the infringement of the entitlement to physical well-being, for instance, through heinous acts such as murder, abuse, torture, rape, and similar transgressions (Honneth & Farrell, 1997). The second level involves understanding oneself as morally accountable. Associated moral injury occurs when others undermine the acknowledgment of an individual’s value in their judgments, often through deceit and fraud (Honneth & Farrell, 1997). The third level concerns confidence in an individual’s own capabilities or values. The corresponding moral injury is humiliation or disparagement, which neglects the recognition of a person’s importance in society (Honneth & Farrell, 1997). The negative emotional reactions that arise from moral injuries, such as feelings of humiliation and anger, serve as the emotional foundation that guides and motivates individuals to struggle for recognition. These negative emotional reactions constitute psychological symptoms. Therefore, individuals realize that their right to social recognition has been unjustly denied.

While some scholars, including Walker, argue that interpreting anger as a response to moral injury is overly individualistic and fails to capture the breadth of anger in social contexts (Walker, 2006), this perspective is open to challenge. Firstly, the notion that anger is rooted in moral injury does not exclude its expression in social settings. Anger toward perceived injustices or violations of moral norms fundamentally reflects a form of moral injury, albeit one that extends beyond the personal to the collective (Nussbaum, 2003). The individualistic interpretation can therefore incorporate social dimensions without losing its core explanatory value. Secondly, the concept of moral injury itself is not confined to personal grievances but encompasses broader social issues (Litz et al., 2009), such as systemic injustice or environmental degradation, which elicit anger as a collective response. Thus, interpreting anger as moral injury addresses both personal and communal manifestations. Furthermore, psychological research highlights the interplay between individual and social factors in shaping emotions, including anger. Social identity theory suggests that individuals derive part of their self-concept from group membership, perceiving harm to the group as personal affronts and triggering anger (Tajfel & Turner, 1978). This aligns with the individualistic interpretation, as the emotional response continues to be anchored in a sense of moral injury, albeit one that is socially constructed and shared.

Moreover, most scholars would agree that anger is a response to unjust behavior (Nussbaum, 2016; Srinivasan, 2018). This explains why most research on anger focuses on its relationship with justice. However, without understanding justice from the perspective of dignity and recognition, explaining why the typical targets of people’s anger are friends or loved ones is challenging. The necessity of justice lies in the vulnerability of individuals and their need for mutual recognition and care. As Habermas states, individuals require protection through mutual understanding because of their susceptibility to harm (Habermas, 1990). This understanding serves a dual purpose: it preserves the integrity of the individual and maintains the crucial bonds of mutual recognition among individuals through which different individuals can stabilize their fragile identities (Habermas, 1990). In other words, justice not only focuses on the autonomous needs of individuals but also on self-respect and mutual recognition among them.

Mutual recognition among individuals is not limited to legal recognition, it also includes the recognition of love. Love is an emotional attachment relationship, most evident within the family, and is manifested in the affectionate attachment between spouses and the emotional bond between parents and children. Honneth submits that love represents the first stage of mutual recognition (Honneth, 1996). When both subjects feel cared for and loved by each other, they recognize their interdependence and mutual needs. Moreover, because needs and emotions can only be met or reciprocated to a certain extent, recognition must involve emotional acknowledgment and encouragement. Therefore, individuals are often more prone to anger toward loved ones compared to strangers because they have a greater attachment to them and yearn for their recognition. In addition to recognition in intimate relationships, there is also legal and value recognition. The angry protests of marginalized people worldwide, the angry voices of feminists, and discontent with populism can all be attributed to the pursuit of equal recognition of marginalized groups.

In sum, anger is a response to perceived intentional harm and is motivated by the desire to gain recognition and respect. It involves seeking acknowledgment of one’s moral worth and importance, demanding equal status for an individual and others, while also expecting respect from others.

## 4. Justified and Unjustified Anger

To evaluate moral emotions, neo-sentimentalism proposes the theory of “appropriateness” of emotions, also known as the Fitting Attitude Theory of Value. According to the neo-sentimentalism perspective, considering something right entails a reasonable positive emotional response toward it, while considering something wrong involves deeming it appropriate to have a negative emotional reaction (Danielsson & Olson, 2007). For instance, Gibbard argues that an action is considered wrong solely when it deserves condemnation, and an action is deemed blameworthy solely when it is reasonable for people to feel resentment toward it (Gibbard, 1990). d’Arms and Jacobson counter that whether an emotion itself is appropriate is one issue, and whether it is correct to feel an emotion in a certain way is a different issue (d’Arms & Jacobson, 2000). The former is an emotional description issue, whereas the latter is a question about the norms of emotions. An appropriate emotion may not necessarily be the correct one, such as when the consequences are dire, although the emotion may be obviously wrong (d’Arms & Jacobson, 2000).

The theory of “appropriateness” of neo-sentimentalism attempts to judge right and wrong in ethics by assessing the rationality of emotions. However, the concepts of appropriateness of emotions and morality are stuck in a circular argument, while d’Arms and Jacobson completely separate the descriptive and normative aspects of emotions. Here, we emphasize that reasonable anger should encompass the “appropriateness” described by d’Arms and Jacobson (d’Arms & Jacobson, 2000), but also include a kind of correctness. However, this correctness is not based on considerations of the consequences of anger, but rather stems from the motivation of desire inherent in anger itself to explore the justification of anger.

The appropriateness of an emotion refers to whether it accurately reflects certain evaluative features of its object and can be considered from two aspects: whether the judgments contained in the emotion accurately reflect the objective situation and, since anger is an emotional blame, whether the extent of the blame should match the severity of the wrongdoing. Regarding the former, if an emotional response is based on false irrational beliefs or does not match the current situation, it is considered inappropriate (Taylor, 1975). Concerning the latter, if a person commits a minor mistake, feeling extremely angry about a small error may be considered inappropriate. Furthermore, anger should be expressed at the right time and in the right circumstances. It should not be prolonged, as this may evolve into resentment. If an individual does not consider the circumstances and expresses anger recklessly, the innocent may be harmed.

Justified anger is based on a reasonable desire for recognition. Before discussing reasonable anger, it is necessary to first discuss the reasonable desire for recognition. The philosophical exploration of recognition can be traced back to Rousseau, who contends that the desire for individual distinction and recognition clashes with the principle of equal respect, attributing society’s detrimental effects to the conflict. In Rousseau’s view, an individual’s desire for recognition corresponds to the pursuit of self-esteem, which relies on others’ approval, fostering comparisons and eroding independence and authenticity. For instance, a wealthy man who is mistaken for a poor person and does not receive the treatment he believes he deserves may feel that his identity is being belittled, even though this treatment does not actually make him truly destitute. He perceives this treatment as an affront to his status. This type of anger is referred to as arrogant anger, which is a response to the subject feeling that their social status has been demeaned. It manifests as a tendency to attack others to elevate oneself (Tanesini, 2021). The self-worth of an arrogant angry person depends on considering themselves superior to certain groups, and when the behavior of others does not highlight their superior status, it may shake their belief in their high value, leading to a sense that their “self-esteem” is being undermined. However, a person with “self-respect” would not easily experience arrogant anger because the values and abilities affirmed by self-respect are not based on comparisons with others. In other words, what is being undermined should be “self-esteem” rather than “self-respect”.

Some philosophers distinguish self-respect from self-esteem (Sachs, 1981; Dillon, 2007; Chazan, 1998; Clucas, 2020). Sachs defines self-respect as a normative concept focused on what one ought to do or believe. (1) It entails recognizing and affirming one’s inherent dignity and worth as a human being, independent of external achievements or failures (Sachs, 1981). Grounded in moral principles, self-respect requires individuals to treat themselves with the same dignity and respect they extend to others. By contrast, self-esteem is a descriptive concept that reflects an individual’s positive or negative evaluation of themselves (Sachs, 1981). It is often contingent on external factors, such as success, approval, or recognition, and fluctuates with achievements and others’ reactions (Sachs, 1981; Dillon, 2007). While self-respect is stable and enduring because it is based on a fundamental recognition of one’s inherent worth, self-esteem is volatile and susceptible to external influences. Sachs argues that self-respect is crucial for moral integrity and ethical behavior, whereas self-esteem, though important for psychological well-being, can foster narcissism or egocentrism when overly reliant on external validation.

A person with self-respect is less prone to arrogant anger, as their values and abilities are affirmed independently of comparisons with others. Conversely, individuals with excessive self-esteem are more likely to exhibit arrogant anger. While low self-esteem is often linked to aggression, studies suggest that high but unstable self-esteem increases vulnerability to aggressive and violent behavior (Baumeister et al., 1996). When their ego is threatened by criticism or rejection, such individuals may react with hostility to protect their self-image—a phenomenon known as “threatened egotism” (Kernis et al., 1989). High self-esteem can also drive social comparison, motivating individuals to compare themselves to others to sustain their positive self-view. While this may inspire improvement, it can also breed envy and resentment when others are perceived as more successful, leading to feelings of inadequacy and a competitive, often toxic, social environment (Alicke & Sedikides, 2009). Furthermore, when self-worth depends entirely on others’ “appraisal respect”, self-esteem becomes extremely fragile. A lack of admiration or recognition can provoke anger rooted in fear, doubt, and concerns about declining social status. However, others’ respect or admiration does not diminish an individual’s intrinsic worth. Self-respect, though also dependent on recognition, arises from mutual acknowledgment among equals and represents a universal right.

The respect related to self-respect is recognition respect, while the respect associated with self-esteem is appraisal respect. Recognition respect is considered a fundamental right owed to all individuals. As stated previously, “To say that persons as such are entitled to respect is to say that they are entitled to have other persons take seriously and weigh appropriately the fact that they are persons in deliberating about what to do” (Darwall, 1977).

Unlike recognition respect, which acknowledges an individual’s inherent dignity and rights as a human being (Galeotti, 2010), appraisal respect involves a positive evaluation of specific virtues, talents, or characteristics (Darwall, 1977). However, not all attributes warrant positive appraisal. Even when a person possesses certain virtues, others are not obligated to esteem those qualities. As Darwall explains, “recognition respect” differs from other attitudes because it can be justified by its object and is formally requested (Darwall, 1977). If someone fails to esteem another’s admirable qualities, they may neglect to provide a deserved response. Nevertheless, esteem, unlike respect for dignity, cannot be expected or demanded. In essence, recognition respect is a moral obligation, while appraisal respect remains discretionary.

Therefore, justified anger arises from the desire for recognition respect—rather than appraisal respect—not being fulfilled. Reasonable anger is based on a desire for recognition of equality, whereas unreasonable anger is a form of arrogant anger that arises when the desire for recognition of superiority is not fulfilled. Specifically, when an individual believes that their social status and worth are superior to others, and this perception is challenged, it can trigger an arrogant anger response.

At the core of arrogant anger lies the feeling that the other person’s actions pose a challenge to one’s status, leading to the interpretation of their behavior as a form of contempt and offense. Anything that threatens the sense of superiority held by an arrogant person will undoubtedly be perceived as a painful injury (Nussbaum, 2021). Arrogant anger is considered unreasonable because people never fully esteem or acknowledge others’ full humanity, but only focus on themselves (Nussbaum, 2021). Arrogant anger ultimately stems from a self-centered perspective. When individuals fixate excessively on their personal needs or those that are unfulfilled, feelings of self-centered anger can surface. Similarly, when others impose similar expectations on themselves that are not embraced, this often reflects a belief that the other party lacks the authority to make such demands. This evaluation of entitlement may fuel self-centered anger and a longing for acknowledgment of one’s superiority.

Why should we distinguish between harming self-respect and harming self-esteem? Specifically, why should we differentiate between recognition respect and appraisal respect? Contemporary recognition theorists argue that recognition is crucial for fostering feelings of self-worth, self-respect, and self-esteem, one of the main reasons for defending recognition. However, when recognition theorists’ concept of self-worth is unclear and does not distinguish between self-esteem and self-respect, it could potentially lead to an extreme understanding in which the actions of others (especially those that do not meet one’s desires or expectations) are attributed as harming one’s self-respect. A pertinent example of this phenomenon is evident in today’s world inundated by identity politics, including various types of resistant anger, such as anti-colonialism, anti-racism, anger struggles of marginalized groups, and so on (Fukuyama, 2018).

In addition, there exist various types of arrogant anger, such as cultural, racial, class, and well-known gender arrogance. One reason for which it is challenging to make valued judgments on political anger is that various desires for recognition are mixed: desires for equal recognition and special recognition may coexist simultaneously. Tanesini attributes the anger of white working-class men in the United States and the United Kingdom to a form of arrogant anger, because their sense of self-worth depends on their social status being superior to that of some members of other social groups (Tanesini, 2021). When their work conditions gradually deteriorate and their previous social superiority over all women and other colored races diminishes, they develop a response of arrogant anger. Nevertheless, this explanation is just one aspect: the anger of white working-class men is also due to the exacerbation of inequality and the contempt and arrogance exhibited by the elite toward them.

In sum, anger arises when the desire for recognition is not fulfilled, leading to a sense of moral injury. However, it is imperative to recognize that not all desires for recognition are reasonable. Therefore, it is crucial to clarify the specific demands for recognition behind the anger and to distinguish between reasonable and unreasonable desires for recognition.

## 5. The Guidance and Transformation of Anger

Anger is often perceived as either a destructive force that fuels violent conflicts or a constructive energy that drives individuals to fight injustice and promote social progress. However, both perspectives are limited. Viewing anger solely as destructive overlooks its role in highlighting injustices and issues that need addressing. Conversely, framing anger only as a symbol of justice can lead to moral judgments under the guise of righteousness. To fully understand anger, it is essential to broaden our perception. By recognizing the personal and societal issues that anger reveals, we can harness its potential to effectively navigate conflicts. This broader understanding transforms anger into a positive force that fosters mutual understanding, respect, and recognition within communities.

First, it is crucial to pay special attention to the emotional injustices experienced by vulnerable and marginalized groups, as well as their demands for recognition, to alleviate their anger and resentment. Today, the anger and passion ignited by identity politics ultimately stem from the pursuit of recognition. As Fukuyama submits, what drives contemporary identity politics is the marginalized groups’ pursuit of equal recognition (Fukuyama, 2018). Furthermore, emotions held by humiliated groups yearning for the restoration of dignity carry more weight than those held by people solely pursuing economic advantages. In conclusion, the combination of economic hardships and feelings of humiliation can sharpen feelings of anger, leading to resentment and hatred among different ethnicities, cultures, and social classes. For instance, individuals experiencing poverty may resent authorities and the wealthy due to economic struggles; marginalized groups may harbor resentment from being overlooked; and the elite class may exhibit arrogance by disregarding the contributions of ordinary people. It is essential to not be entrapped in the anger provoked by identity politics, but rather to follow the guidance of these emotions to resolve conflicts. Moreover, humility—not arrogance—should be maintained, as the success of the elite relies on cooperation within society, as they are part and beneficiaries of society. Regardless of cultural background or social status, we are all members of the human community. Therefore, the purpose of anger should not lead to division but guide us toward mutual recognition, respect, and justice, ultimately building a better community for all.

Certainly, when experiencing feelings of anger in everyday interpersonal relationships, we should follow the guidance of anger to comprehend our deepest desires and beliefs, as well as those of others. People often lack a sufficient understanding of themselves and others. It is precisely because “knowing yourself” is extremely challenging that it becomes the maxim of the Delphic Oracle. Understanding one’s own desires and beliefs undoubtedly forms a crucial part of “knowing yourself”, and spontaneous and genuine emotional expressions often directly reflect one’s desires and beliefs. Only when we comprehend our desires and beliefs can we examine them critically, distinguishing between necessary and unnecessary desires and authentic and illusory beliefs. In the unfortunate event we become the target of someone else’s anger, we can gain clearer insight into the desires of others, what they cherish, and their expectations through anger. This way, we can avoid responding to anger with more anger, resist being incited toward hatred by others’ words, and refrain from falling into the trap of simplistic fallacies: believing that our anger is just and reasonable while dismissing others’ as irrational and extreme. It is only through understanding oneself and others that we can achieve effective communication and mutual understanding in interpersonal relationships. Again, it is crucial to cultivate a sense of trust and friendship among citizens. Whether victims of injustice or targets of others’ anger, responding with violence to dissatisfaction should be avoided. Efforts should instead be made to foster friendship and cooperation with others. Nussbaum posits that (Nussbaum, 2021) “If we cannot form cooperations with well-intentioned people on the “other side”—not in blind trust, but after careful scrutiny—we have no hope of eventual reconciliation”. Furthermore, the fiery anger of identity politics does not necessarily lead to divisions among races, classes, or cultures. It is essential to transcend primal identities based on nationality, skin color, religion, class, gender, and sexual orientation and build a broader community of friendships. Building trust and citizenship on a larger scale is crucial, as outlined by Fukuyama, laying the foundation for more extensive and meaningful value recognition (Fukuyama, 2018). This should be the direction and goal for contemplation.

Finally, it is essential to regulate and guide emotional expression in today’s online society. The expression of anger in online communities often descends into emotional outbursts, transforming into malicious insults, verbal abuse, and attacks that perpetuate mutual animosity among different groups, further leading to the fragmentation of societal unity. To prevent societal division caused by the unchecked proliferation of angry emotions in the online sphere and to promote unity and prosperity, efforts from the state, society, and individuals should be combined to govern and prevent the spread of anger in the online realm. The state should establish regulations and laws governing emotional expression online to ensure that the Internet does not become a lawless space for violent emotional outbursts. Additionally, supervision and control should be exercised over Internet platforms to effectively monitor negative emotions in the online society, enabling better intervention, guidance, and resolution. Various organizations within society should collaborate to foster a clean and healthy online social environment, collectively resisting the spread of low-quality information and enhancing self-purification capabilities regarding information. Individual citizens should enhance their personal integrity, improve their ability to discern types of anger, remain vigilant against being incited by information and emotions online, and refrain from engaging in online violence and outbursts.

## Data Availability

No new data were created or analyzed in this study.
